# Supplementary material for: PDE6D Mediates Trafficking of Prenylated Proteins NIM1K and UBL3 to Primary Cilia
Source: Cells. 2023 Jan 13;12(2):312. doi: 10.3390/cells12020312 (PMC9857354; doi:10.3390/cells12020312)
Supplement: Supplementary file 1 [file cells-12-00312-s001.zip › Supplementary_Materials.pdf]

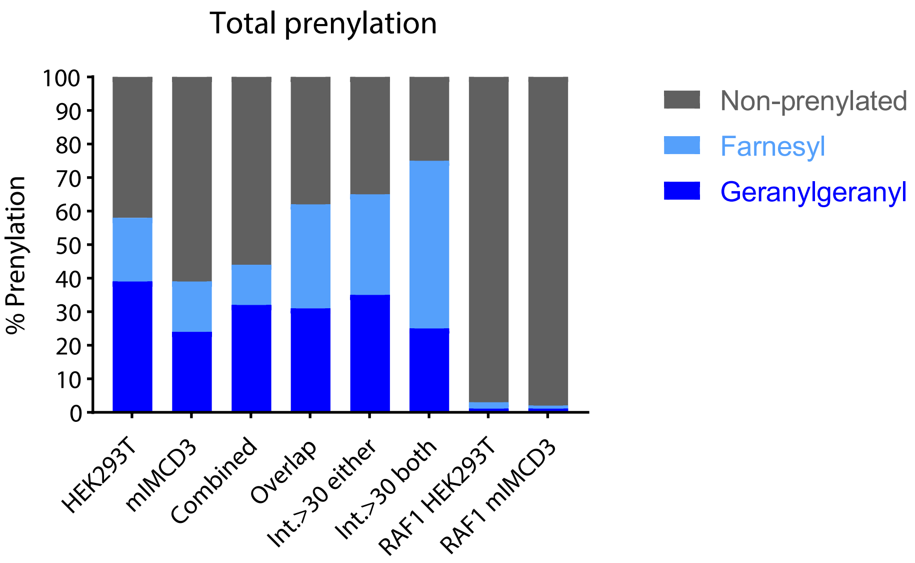

Figure S1. Quantification of prenylation percentage of potential PDE6D interactors. Potential PDE6D interactors categorized based on prenylation moiety in different (sub)datasets, with protein complexes not considered as one entity. Bars indicated with 'HEK293T' and 'mIMCD3' represents individual datasets performed in HEK293T and mIMCD3 Flp-in cells, respectively. Bar indicated with 'Combined' represents the complete set of unique proteins (including proteins present in both datasets) resulting from combining both datasets. Bar indicated with 'Overlap' represents all proteins that are present in both datasets. Bars indicated with 'Int.>30' represent proteins that have an LFQ intensity score above 30 in at least one dataset ('either') or in both datasets ('both'). Bars indicated with 'RAF1 HEK293T' and 'RAF1 mIMCD3' show the prenylation percentage in the control. Percentage of geranylgeranylated proteins is indicated in dark blue, percentage of farnesylated proteins is indicated in light blue, and percentage of non-prenylated proteins is indicated in dark grey.

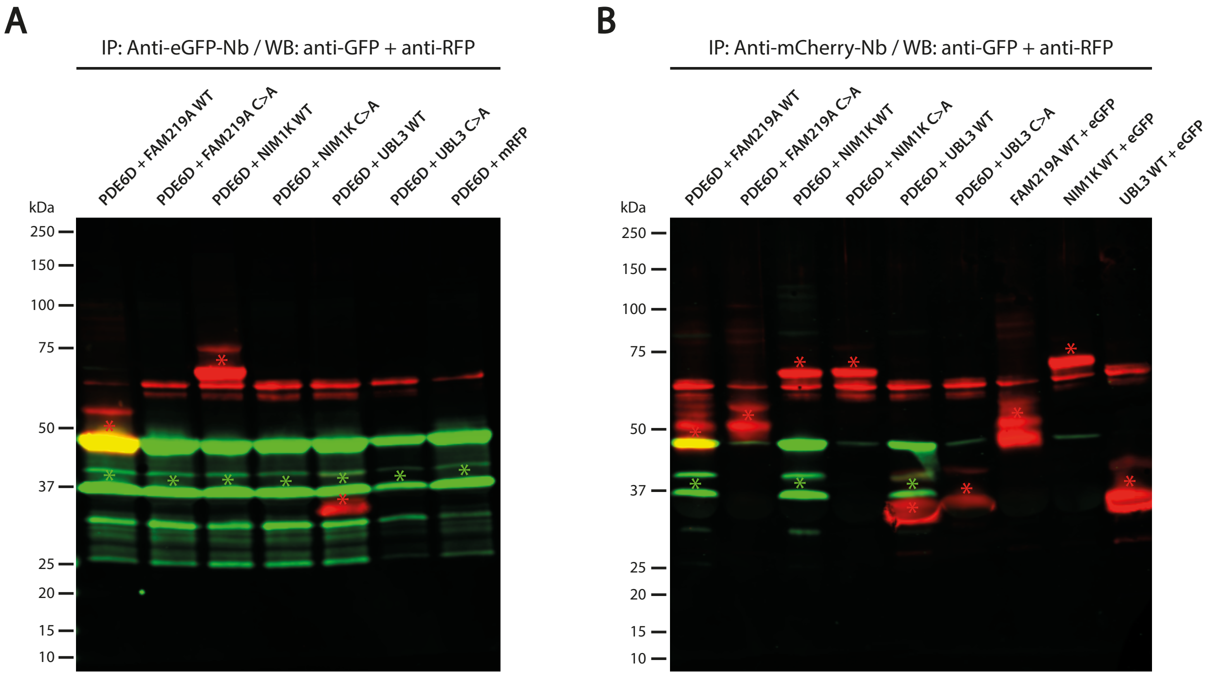

Figure S2. Confirmation of VIP assay by western blot. (**A**) Western blot showing the immunoprecipitation of eGFP-tagged PDE6D (indicated by green asterisks) by anti-eGFP nanobody beads (nb) followed by anti-GFP and anti-RFP antibody staining. Co-immunoprecipitated proteins, including FAM219A WT, NIM1K WT, and UBL3 WT, are indicated by red asterisks. (**B**) Western blot showing the immunoprecipitation of mRFP-tagged FAM219A WT/C>A, NIM1K WT/C>A, and UBL3 WT/C>A (indicated by red asterisks) by anti-mCherry nanobody beads (nb) followed by anti-GFP and anti-RFP antibody staining. Co-immunoprecipitated eGFP-tagged PDE6D, is indicated by green asterisks. Of note: For the WT forms of FAM219A and UBL3 two bands were detected, while for the C>A mutants only the upper band was detected. This could be due to the maturation and prenylation of the WT forms.

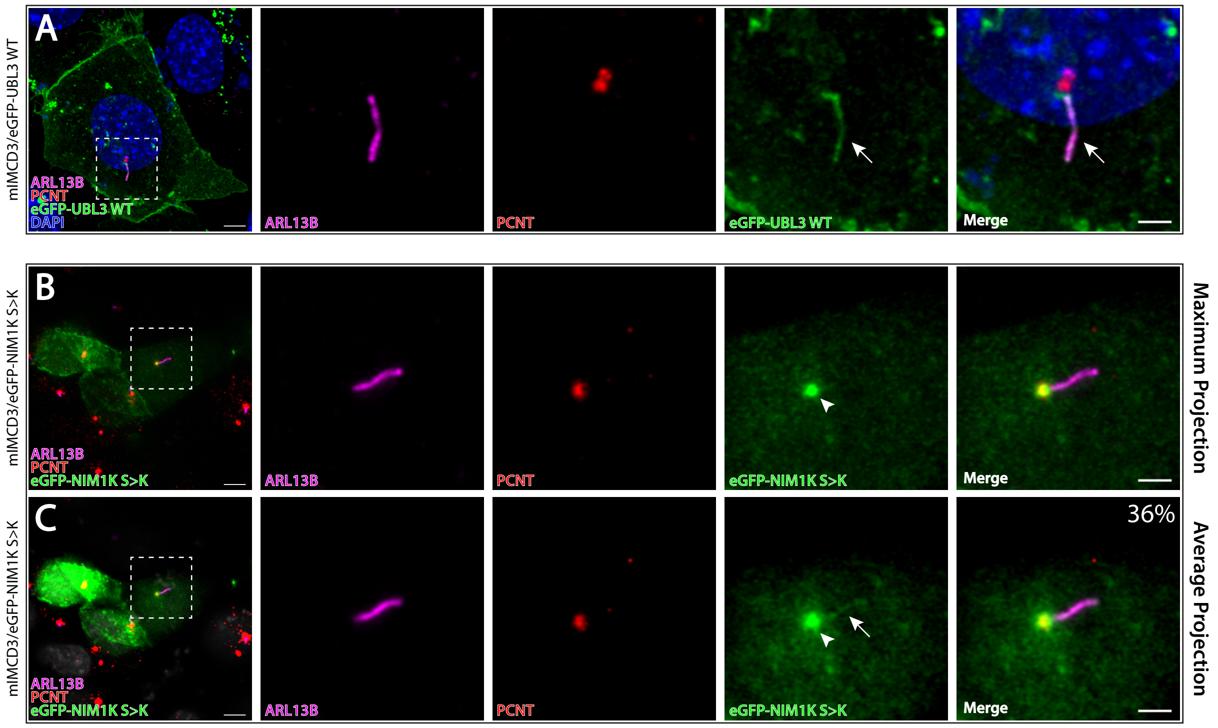

Figure S3. Localization of eGFP-UBL3 WT expressed in mIMCD3 cells. (**A**) Ciliogenesis induced mIMCD3 cells transfected with eGFP-UBL3 WT. Cilia were stained with antibodies against ARL13B (magenta). Basal bodies were stained with antibodies against Pericentrin (PCTN, red). Nuclei were stained with DAPI (blue). Arrow indicates eGFP-UBL3 WT staining inside the cilium. Scale bar: 5 µm (inset: 2 µm). (**B-C**) Comparison of maximum projection and average projection of Z-stacks for eGFP-NIM1K S>K expressed in mIMCD3 cells. Percentages indicated in (**C**) show the percentage of transfected ciliated cells that show both a ciliary localization and a basal body localization.

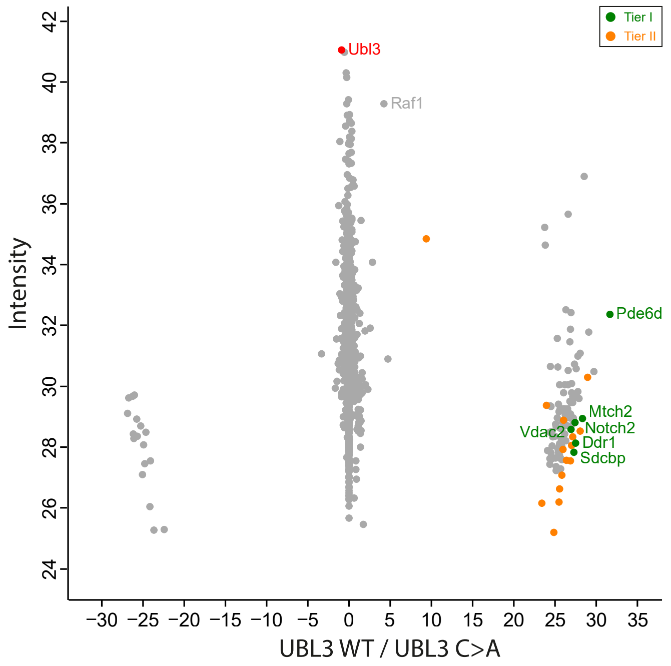

Figure S4. Identification and clustering of potential UBL3 interactors. Scatterplot showing enriched proteins comparing UBL3 WT to UBL3 C>A in mIMCD3 Flp-in cells. Bait protein is shown in red. Significantly enriched proteins (UBL3 WT vs. RAF1 + UBL3 C>A) are indicated in green (Tier I) and orange (Tier II, with Tier I and Tier II proteins determined by FDR≤ 0.05 and p≤0.05, respectively. *X*-axis represents log2 ratio between UBL3 WT and UBL3 C>A. *Y*-axis represents label-free quantification (LFQ) intensity score, indicating the relative amount of proteins in the dataset.

Table S1. Protein list of potential PDE6D interactors. (**Tab 1**) Protein list of potential PDE6D interactors from HEK293T cells. Bait protein is shown in red. Significantly enriched geranylgeranylated proteins are indicated in dark blue and significantly enriched farnesylated proteins are indicated in light blue. Significantly enriched non-prenylated proteins are indicated in dark grey. Proteins that form protein complexes, including a geranylgeranylated guanine nucleotide-binding protein (G protein) and a geranylgeranylated SCF (SKP1-CUL1-F-box protein) ubiquitin ligase complex are indicated with * and # respectively. (**Tab 2**) Protein list of potential PDE6D interactors from mIMCD3 cells. Bait protein is shown in red. Significantly enriched geranylgeranylated proteins are indicated in dark blue and significantly enriched farnesylated proteins are indicated in light blue. Significantly enriched non-prenylated proteins are indicated in dark grey. Proteins that form protein complexes, including a geranylgeranylated guanine nucleotide-binding protein (G protein), a geranylgeranylated SCF (SKP1-CUL1-F-box protein) ubiquitin ligase complex, and a non-prenylated COP9 signalosome (CSN) complex are indicated with *, #, and ^ respectively. (**Tab 3**) Complete set of unique proteins (including proteins present in both datasets) resulting from combining both the HEK293T and mIMCD3 datasets. Colors and protein complexes are indicated identical, as described above. (**Tab 4**) All proteins that are present in both datasets. Colors and protein complexes are indicated identical, as described above. (**Tab 5-6**) Proteins that have an LFQ intensity score above 30 in at least one dataset ('either') or in both datasets ('both'). Colors and protein complexes are indicated identical, as described above. (**Tab 7**) Prenylation percentage calculations represented in Figure 1C and Figure S1.

Table S2. Protein list of potential RAF1 interactors. (**Tab 1**) Protein list of potential RAF1 interactors from HEK293T cells. Bait protein is shown in red. Significantly enriched geranylgeranylated proteins are indicated in dark blue and significantly enriched farnesylated proteins are indicated in light blue. Significantly enriched non-prenylated proteins are indicated in dark grey. Several proteins that form protein complexes are indicated with &, $, /, >, %, ~, <. (**Tab 2**) Protein list of potential RAF1 interactors from mIMCD3 cells. Bait protein is shown in red. Significantly enriched geranylgeranylated proteins are indicated in dark blue and significantly enriched farnesylated proteins are indicated in light blue. Significantly enriched non-prenylated proteins are indicated in dark grey. Several proteins that form protein complexes are indicated with /, }.

Table S3. Protein list of potential UBL3 interactors and GetGo analysis. (**Tab 1**) Protein list of potential UBL3 interactors from mIMCD3 Flp-in cells. Bait protein is shown in red. Significantly enriched proteins (UBL3 WT vs. RAF1 + UBL3 C>A) are indicated in green (Tier I) and orange (Tier II), with Tier I and Tier II proteins determined by FDR≤0.05 and p≤0.05, respectively. (**Tab 2**) GetGo analysis of potential UBL3 interactors, including Tier I and Tier II proteins.
